# Emergency Department Use Following Self‐Harm and Suicide Ideation: An Analysis of the Influence of Cultural and/or Linguistic Diversity Using Data From the Self‐Harm Monitoring System for Victoria (2012–2019)

**DOI:** 10.1111/inm.13411

**Published:** 2024-09-09

**Authors:** Gowri Rajaram, Jo Robinson, Lu Zhang, Katrina Witt

**Affiliations:** ^1^ Orygen Parkville Victoria Australia; ^2^ Centre for Youth Mental Health The University of Melbourne Parkville Victoria Australia; ^3^ Department of Psychiatry, Melbourne Neuropsychiatry Centre The University of Melbourne Carlton Victoria Australia

**Keywords:** culturally and linguistically diverse (CALD), emergency department, help‐seeking behaviour, self‐harm, suicide ideation

## Abstract

Self‐harm and suicide ideation are global health concerns, significantly impacting culturally and linguistically diverse (CALD) populations. Emergency departments (EDs) play a role in intervening following such presentations, yet there is limited research focusing on the CALD population's use of these services in Australia. This study aimed to explore patterns in ED use for self‐harm and suicide ideation, comparing CALD and non‐CALD persons in terms of service use, presentation themes and likelihood of repeat presentations. This was a cross‐sectional analysis of data from presentations for self‐harm and suicide ideation to the ED of a major metropolitan hospital in Victoria, Australia, from 2012 to 2019. The study used thematic analysis of triage notes, recurrent event analysis and logistic and linear regressions to compare CALD and non‐CALD presentations. CALD presentations comprised 1.3% (*n* = 202) of the total (*n* = 15 606). CALD presentations were more likely to occur during business hours, less likely to be triaged as urgent and more likely to result in ward admission. Occupation stressors were more common in CALD presentations. A lower likelihood of repeat presentations was observed among CALD persons. The study also highlighted the limitations of current data collection practices in capturing the full spectrum of CALD presentations. This study found variability in the recording of CALD status, warranting further investigation into how data collection in EDs may be improved. Increased ward admission rate and lower likelihood of repeat presentation by CALD persons also indicate that further research is required to understand help‐seeking and clinical decision‐making in the CALD population.

## 1 Introduction

Suicide is the leading cause of death in Australians aged 15–44 years (Australian Institute of Health and Welfare 2023a). Self‐harm, defined as intentional self‐poisoning or self‐injury irrespective of type of motive or the extent of suicidal intent (Hawton et al. 2003), is more common and represents a global health concern which disproportionately impacts young people and females (Carr et al. 2016; Arensman, Corcoran, and Mcmahon 2018). Suicide ideation is even more common. Data from the National Study of Mental Health and Wellbeing (Australian Bureau of Statistics 2020–21) indicate that 3.4% of Australians aged 16–85 years have experienced suicide ideation in the past year, with 2.0% engaging in self‐harm in the same period.

Emergency departments (EDs) are often a primary point of contact for people who have self‐harmed or thought about suicide (Zanus et al. 2017) and play a role in evaluating safety and imminent risk, then triaging to appropriate levels of care which may include inpatient hospitalisation or referrals elsewhere (Asarnow, Babeva, and Horstmann 2017). Mental health nurses are often the first to provide therapeutic care within this setting. In Australia, the rate of self‐harm hospitalisations has remained consistent over the past decade, from 120.6 in 2011–2012 to 104.6 per 100 000 in 2021–2022, indicating that there has been a steady need for targeted mental health interventions in this setting (Australian Institute of Health and Welfare 2023b).

The culturally and linguistically diverse (CALD) population is both large and heterogenous: Half of all Australians are either first‐ or second‐generation migrants, including asylum seekers and refugees (Australian Bureau of Statistics 2021). Victoria is Australia's most culturally diverse state (Victorian Department of Health 2022). Furthermore, Australia has a significant international student population (Australian Department of Education 2023). Recognising the diverse make‐up of people who use these services, a responsive healthcare system must capitalise on EDs as intervention points, especially given the preference for EDs over general practitioners (GP) by non‐English speaking populations (Mahmoud, Eley, and Hou 2015). Furthermore, mental health nurses who work in EDs must be equipped to identify and address the potentially unique needs of this population.

Australian evidence suggests that the prevalence of self‐harm and suicide ideation may differ between the CALD and non‐CALD populations. Compared to the non‐CALD population, the CALD population experiences lower rates of suicide (Maheen and King 2023). In males, many ethnic minority groups such as those from South and Eastern Europe, Middle Eastern and Southern Asian backgrounds have significantly lower odds of reporting lifetime suicide ideation but with less pronounced differences for lifetime suicide attempt (Maheen, Haregu, and Armstrong 2024). Globally, there are variations in the epidemiology of self‐harm in specific CALD subpopulations. For example, there is some evidence that younger migrants' experience has higher rates of self‐harm compared to their non‐migrant counterparts in the United States and Germany (Borges et al. 2011; Donath et al. 2019; Plener et al. 2015). While Australian research indicates that the prevalence of both self‐harm and suicide ideation may be lower in CALD populations, this population may experience unique risks relating to self‐harm, mental health and underuse of clinical services. Several factors, including migration‐ and acculturation‐related stressors, as well as societal attitudes towards CALD persons may contribute to this (Murray, Davidson, and Schweitzer 2008; Young and Gordon 2016). Current data indicate that, in Australia, the Australian‐born population experiences the higher rates of self‐harm hospitalisations (141 per 100 000) than the migrant population (42.8 per 100 000) (Pham et al. 2023b) and are 42% more likely to have repeat hospital presentations than the migrant population (Stapelberg et al. 2020). However, international evidence suggests that factors such as stigma (Flink et al. 2014), lack of awareness of clinical services (Gondek and Kirkbride 2018) and a preference for informal supports (Leung 2020) may influence the observed help‐seeking and engagement with clinical services.

CALD populations have been identified as a priority group for research (Colucci, Too, and Minas 2017), policy (Suicide Prevention Australia 2021) and by Royal Commission into Victoria's Mental Health System (State of Victoria 2021). However, there is a lack of suicide prevention research focussing on this specific population (Bowden, Mccoy, and Reavley 2020).

Understanding how people of CALD backgrounds use EDs following self‐harm or suicide ideation is an important step towards providing an evidence base for nurses and other practitioners to guide culturally sensitive care and tailored follow‐up care plans. The aims of this study are to:

1. Identify what proportion of self‐harm and/or suicide ideation presentations to a major metropolitan hospital are by CALD persons;
2. Examine how CALD identity is classified in the triage notes;
3. Examine psychosocial factors recorded in triage notes relating to the presentation;
4. Compare CALD and non‐CALD presentations on the nature of service use during and after initial presentation;
5. Compare rates of re‐presentation between CALD and non‐CALD persons.

## 2 Methods

### 2.1 Study Design and Setting

This was a cross‐sectional study of self‐harm and suicidal ideation‐related presentations to the ED of a major metropolitan hospital between 12 January 2012 and 31 December 2019. The catchment area of the study hospital is diverse with proportions of CALD persons that are greater than the national average (Robinson et al. 2020).

### 2.2 Data Sources and Case Ascertainment

Data were sourced from self‐harm monitoring system for Victoria electronic medical records, and additional coding was undertaken to identify self‐harm and suicidal ideation‐related attendances. This system collects ED data for presentations related to self‐harm or suicide ideation by persons aged 9 and above, and for whom the ED presentation represents the first help‐seeking occasion for that episode. The case ascertainment process has been described elsewhere (Robinson et al. 2020). In brief, presentations were coded by pairs of trained research assistances to determine instances of self‐harm or suicide ideation. The self‐harm monitoring system for Victoria has received ethical approval from the Melbourne Health Human Research Ethics Committee (HREC; 2017.342).

### 2.3 Study Variables

Variables used in this study, provided in detail in Table S1, include demographic characteristics such as gender and age, as well as presentation characteristics such as mode and time of arrival, triage category ranging from 1 or 2 (life‐threatening) to 4 or 5 (potentially serious or less urgent) (Australasian College for Emergency Medicine 2023), length of ED stay and disposition. Presentations were linked to the same person via their hospital‐specific medical record number (MRN), allowing for survival analyses and coding of CALD status. Postcodes recorded for each presentation were used to ascertain index of relative socioeconomic advantage and disadvantage (Australian Bureau of Statistics 2018).

Gender/sex was considered a binary variable (male or female). In this study, the term ‘gender/sex’ was used to categorise presentations due to variations in the source data, and presentations were further classified as CALD or non‐CALD. The study population was also divided into young people (<25 years) and adults (25+ years) to be able to report on the demographic make‐up of the sample (see Table 1). Age was included as a continuous variable in analyses.

**TABLE 1:** Demographic characteristics, psychosocial and economic factors, and presentation characteristics by CALD status.

Demographic characteristics	CALD	Non‐CALD	Total	OR (95% CI)	*p*	aOR^a^ (95% CI)	*p*
Age group							
Young people	54 (26.7)	4477 (29.1)	4531 (29.0)	0.89 (0.65–1.21)	0.469		
Adults (reference)	148 (73.3)	10 927 (70.9)	11 075 (71.0)				
Gender							
Female (reference)	88 (43.6)	7890 (51.2)	7978 (51.1)				
Male	114 (56.4)	7464 (48.5)	7578 (48.6)	1.37 (1.03–1.81)	0.028		
Other	0 (0.0)	50 (0.3)	50 (0.3)	—	—		

	Median (IQR)	Median (IQR)	Median (IQR)				
Index of relative socioeconomic advantage and disadvantage, decile	8 (6–8)	8 (6–9)	8 (6–9)	0.95 (0.89–1.00)	0.053		

Psychosocial and economic factors	CALD	Non‐CALD	Total	OR (95% CI)	*p*	aOR (95% CI)	*p*
Clinical							
Suicide history	28 (13.9)	2300 (14.9)	2328 (13.9)	0.92 (0.61–1.37)	0.672	0.95 (0.63–1.42)	0.793
Mood disorders, including symptoms of mood disorders	66 (32.7)	5900 (38.3)	5966 (38.2)	0.78 (0.58–1.05)	0.103	0.80 (0.60–1.08)	0.145
Anxiety disorders	19 (9.4)	2206 (14.3)	2225 (14.3)	0.62 (0.39–1.00)	0.049	0.64 (0.40–1.04)	0.071
Trauma and stress‐related disorders, including symptoms of trauma and stress‐related disorders	5 (2.5)	823 (5.3)	828 (5.3)	0.45 (0.18–1.10)	0.079	0.47 (0.19–1.15)	0.097
Psychotic disorders, including symptoms of psychotic disorders	21 (10.4)	1995 (13.0)	2016 (12.9)	0.78 (0.50–1.23)	0.283	0.75 (0.47–1.18)	0.211
Changes and/or problems relating to medication	11 (5.5)	693 (4.5)	704 (4.5)	1.22 (0.66–2.26)	0.520	1.21 (0.66–2.24)	0.535
History of and/or current alcohol and/or drug use	20 (9.9)	4251 (27.6)	4271 (27.4)	0.29 (0.18–0.46)	<0.001	0.27 (0.17–0.43)	<0.001
Violence and aggression risk	10 (5.0)	741 (4.8)	751 (4.8)	1.03 (0.54–1.95)	0.926	1.00 (0.52–1.89)	0.989
Social							
Problems related to housing and living circumstances, including homelessness	10 (5.0)	561 (3.7)	571 (3.7)	1.38 (0.73–2.62)	0.327	1.31 (0.69–2.49)	0.411
Problems with personal relationships	19 (9.4)	1130 (7.3)	1149 (7.4)	1.31 (0.81–2.11)	0.264	1.35 (0.84–2.18)	0.219
Occupational or financial stressors (including study and work)	8 (4.0)	250 (1.6)	258 (1.7)	2.50 (1.22–5.13)	0.012	2.45 (1.19–5.02)	0.015
Service use							
General practitioner	6 (3.0)	236 (1.5)	242 (1.6)	1.97 (0.86–4.48)	0.107	2.01 (0.88–4.58)	0.096
Police involvement and/or treatment order (excl. mental health act)	13 (6.4)	754 (4.9)	767 (4.9)	1.34 (0.76–2.36)	0.316	1.27 (0.72–2.23)	0.402
Transported under Mental Health Act	41 (20.3)	3079 (20.0)	3120 (20.0)	1.02 (0.72–1.44)	0.913	1.00 (0.71–1.41)	0.984

Presentation characteristics	CALD	Non‐CALD	Total	Odds ratio	*p*	Adjusted odds ratio	*p*
Reason for presentation							
Suicide ideation	131 (64.9)	7724 (50.1)	7855 (50.3)	—	—	—	—
Self‐harm	71 (35.2)	7680 (49.9)	7751 (49.7)	0.55 (0.41–0.73)	<0.001	0.60 (0.45–0.82)	0.001
Time of presentation							
Early morning (01:00–08:59)	31 (15.4)	2972 (19.3)	3003 (19.2)	0.57 (0.38–0.86)	0.007	0.57 (0.37–0.88)	0.0010
Business hours (09:00–16:59) (reference)	94 (46.5)	5140 (33.4)	5234 (33.5)	—	—	—	—
Night (17:00–00:59)	77 (38.1)	7292 (47.3)	77 (38.1)	0.58 (0.43–0.78)	<0.001	0.57 (0.42–0.79)	0.001
Arrival mode							
Self‐present	34 (16.8)	2999 (19.5)	3033 (19.4)	0.83 (0.57–1.22)	0.339	0.79 (0.53–1.18)	0.251
Ambulance (reference)	123 (60.9)	9004 (58.5)	9127 (58.5)	—	—	—	—
Police	16 (7.9)	1138 (7.4)	1154 (7.4)	1.03 (0.61–1.74)	0.914	0.87 (0.49–1.53)	0.622
Other	29 (14.4)	2215 (14.4)	2244 (14.4)	0.96 (0.64–1.44)	0.838	0.75 (0.48–1.17)	0.210
Triage category							
1 or 2	14 (6.9)	1968 (12.8)	1982 (12.7)	0.52 (0.30–0.90)	0.018	0.50 (0.27–0.92)	0.027
3 (reference)	142 (70.3)	10 286 (66.8)	10 286 (66.8)	—	—	—	—
4 or 5	46 (22.8)	3150 (20.5)	3196 (20.5)	1.06 (0.76–1.48)	0.742	1.06 (0.75–1.50)	0.725
Receipt of emergency department assessment							
Doctor	195 (96.5)	14 764 (95.9)	14 959 (98.9)	1.21 (0.57–2.58)	0.626	1.02 (0.48–2.19)	0.956
Mental health	144 (71.3)	9873 (64.1)	10 017 (64.2)	1.39 (1.02–1.89)	0.035	1.31 (0.94–1.81)	0.109
Destination							
Home (reference)	70 (34.7)	6194 (40.2)	6264 (40.1)	—	—	—	—
Ward	27 (13.4)	1794 (11.7)	1821 (11.7)	1.33 (0.85–2.00)	0.209	1.79 (1.08–2.94)	0.023
Mental health unit	28 (13.9)	1156 (7.5)	1184 (7.6)	2.14 (1,38–3.34)	0.001	2.16 (1.36–3.44)	0.001
Observation unit	63 (31.2)	4845 (31.5)	4908 (31.5)	1.15 (0.82–1.62)	0.422	1.34 (0.93–1.93)	0.110
Left before treatment completion	11 (5.5)	1304 (8.5)	1315 (8.4)	0.75 (0.39–1.41)	0.369	0.75 (0.37–1.52)	0.424
Other	3 (1.5)	111 (0.7)	114 (0.7)	2.39 (0.74–7.71)	0.144	2.84 (0.87–9.31)	0.085

	Median (IQR)	Median (IQR)	Median (IQR)	Crude coefficient	*p*	Coefficient	*p*
Time intervals							
Emergency department length of stay, hours	6.0 (3.1–13.2)	4.8 (2.7–9.9)	4.8 (2.7–9.)	1.78 (0.80–2.76)	<0.001	2.18 (1.17–3.19)	<0.001
Time to mental health assessment, hours	1.9 (0.8–3.8)	1.7 (0.7–3.6)	1.72 (0.72–3.6)	−0.88 (−7.63–5.86)	0.797	−1.00 (−8.30–6.30)	0.787

^a^ Adjusted for age, gender, IRSAD decile, time of presentation, reason for presentation.

### 2.4 Analysis of Triage Notes

#### 2.4.1 CALD Status

CALD status was ascertained by manually coding evidence of likely CALD status contained in the free‐text notes created by triage nursing staff on presentation using Microsoft Excel. People were determined as being of CALD background if the triage comment indicated that the individual either speaks a language other than English as their primary language, or if they were born in a non‐English speaking country, consistent with the definition recommended by Pham et al. (2021). Due to the variability of language used in triage notes, a deductive approach using a predefined criteria was used to ensure internal consistency and reproducibility; these incorporated data item standards identified in the Victorian Family Violence Data Collection Framework (State of Victoria 2019). CALD status was also considered a static characteristic. Thus, where an individual had made several presentations during the study period, identified by their hospital unit record number, CALD status was applied to all presentations. Presentations where no indicators of CALD status were present in the triage comment comprised the non‐CALD group.

#### 2.4.2 Psychosocial and Economic Factors

Inductive analysis was used to identify common psychosocial and economic factors across presentations. This method allows replicable and valid inferences to be made from data, with an aim to generate concepts or categories describing phenomenon identified in the data (Elo and Kyngäs 2008). The process by which factors were coded in the triage comments involved preparation, organising and reporting. A coding framework was prepared and used for manual coding of factors, pilot tested by two independent coders (Figure S1). Interrater reliability for the items in the coding framework ranged from 0.74 to 0.98. Items with an interrater reliability rating below 0.61 were excluded from subsequent analyses.

### 2.5 Statistical Analyses

#### 2.5.1 Analysis of Service Provision

Descriptive statistics, including frequencies and percentages, were used to examine categorical, continuous and binary measures of service provision and utilisation listed in Table S1. Linear regression was used to examine differences in continuous outcomes between the CALD and non‐CALD group, while logistic regression was used for categorical outcomes. The regression models were adjusted for age group, gender, socioeconomic status and whether the presentation was for self‐harm or suicide ideation. The categorical variable of timing of presentation (business hours, night, early morning) was also included in the regression models to account for differences in staffing and resource provision.

#### 2.5.2 Recurrent Event Analysis

To examine the likelihood of re‐presentation for self‐harm and/or suicidal ideation within the study period (2012–2019), two forms of survival analysis were conducted. First, a Cox proportional hazards model was used to determine the likelihood of first re‐presentation for self‐harm and/or suicide ideation, and Kaplan–Meier curves were plotted to illustrate the difference between the CALD and non‐CALD group in their time to first re‐presentation for self‐harm and/or suicide ideation. To analyse all presentations by every individual, recurrent event analysis was also conducted using the Andersen–Gill (A–G) extension of the Cox proportional hazards model. First, a univariate model was first fitted for each predictor variable (gender, age, CALD status, socioeconomic status). Then, a multivariate model was fitted to include all predictor variables simultaneously. Concordance, Wald statistic and likelihood ratio test are reported in Table 2. The follow‐up period for the study was defined as time since index presentation, and all data were censored on 31 December 2019.

**TABLE 2:** Model fit statistics for A–G model.

Model	Model fit statistic	Value	Standard error	Degrees of freedom	*p*
AG	Concordance	0.543	0.004		
	Likelihood ratio	216		5	<0.001
	Wald	248		5	<0.001
	Score (logrank)	276.4		5	<0.001
	Robust	182.8			<0.001

## 3 Results

### 3.1 Characteristics of the Study Population

The study population comprised 15 606 self‐harm and suicide ideation presentations between 2012 and 2019. CALD presentations were characterised by a higher proportion of males compared to the non‐CALD group, while both the CALD and non‐CALD group were similar in terms of socioeconomic status and age distribution (Table 1).

### 3.2 Ascertainment of CALD Status

Following manual coding of the data set, 158 presentations (1.0% of the total sample) were identified as presentations by CALD persons. Language‐related indicators were present in half of all CALD‐identified presentations (*n* = 83, 52.5%). As we considered CALD status as a static characteristic, we applied CALD status to all presentations within the study population with an MRN that matched the CALD‐identified presentations. This process identified an additional 44 presentations, resulting in a total CALD group of 202 presentations (1.3% of the total sample) made by 150 CALD persons.

### 3.3 Psychosocial and Economic Factors

Overall, 13.9% (*n* = 2328) of presentations noted a history of self‐harm or suicide ideation prior to the index presentation (Table 1). There were few differences between CALD and non‐CALD presentations. The CALD group were 73% less likely than the non‐CALD group to report current or past alcohol and/or drug use (aOR = 0.27, 0.17–0.43, *p* < 0.001) and were more than twice as likely to report occupational or financial stressors (aOR = 2.45, 1.19–5.02, *p* = 0.015).

### 3.4 Characteristics of Emergency Department Use

Overall, the reason for presentation was evenly split between self‐harm (*n* = 7751, 49.7%) and suicide ideation (7855, 50.3%) over this 8‐year period (Table 1). However, presentations by CALD people were more often for suicide ideation (*n* = 131, 64.9%) than for self‐harm (*n* = 71, 35.2%) (Table 2).

Almost half of all presentations occurred during the night (*n* = 7369, 47.2%). Presentations by CALD people were more likely to occur during business hours (*n* = 94, 46.5%) than presentations by non‐CALD people (*n* = 5140, 33.4%).

Approximately two‐thirds of presentations were triaged as urgent according to the Australasian Triage Scale classifications (*n* = 10 428, 66.8%), and the CALD group were half as likely as the non‐CALD group to be triaged at categories 1 (immediately life‐threatening) or 2 (imminently life‐threatening or important time‐critical treatment or very severe pain). Almost three‐quarters (*n* = 144, 71.3%) of the CALD group received a mental health assessment, compared to 64.1% (*n* = 9873) of the non‐CALD group.

In terms of destination after the ED, people were most commonly discharged home (*n* = 6264, 40.1%), or to an observation unit (*n* = 4908, 31.5%). The CALD group was more likely than the non‐CALD group to be admitted to a general medical/surgical ward (13.4% vs. 11.7%, aOR 1.79, 95% CI 1.08–2.94, *p* = 0.023) and more likely to be sent to a mental health unit (13.9% vs. 7.5%, aOR 2.16 (1.36–3.44), *p* = 0.001).

### 3.5 Recurrent Event Analysis

The majority of those in the study population had only one presentation recorded during the study period (*n* = 7754, 79.2%). Around 1 in 10 had two presentations (*n* = 1127, 11.5%), while just under 1 in 10 (*n* = 916, 9.35%) had three or more presentations. To examine the likelihood of representation, two analytical approaches were used. The first, using survival analysis to examine differences in first repeat presentation, and the second using the Anderson–Gill extension of the Cox proportional hazards model.

### 3.6 Survival Analysis and First Repeat Presentation

**FIGURE 1:**
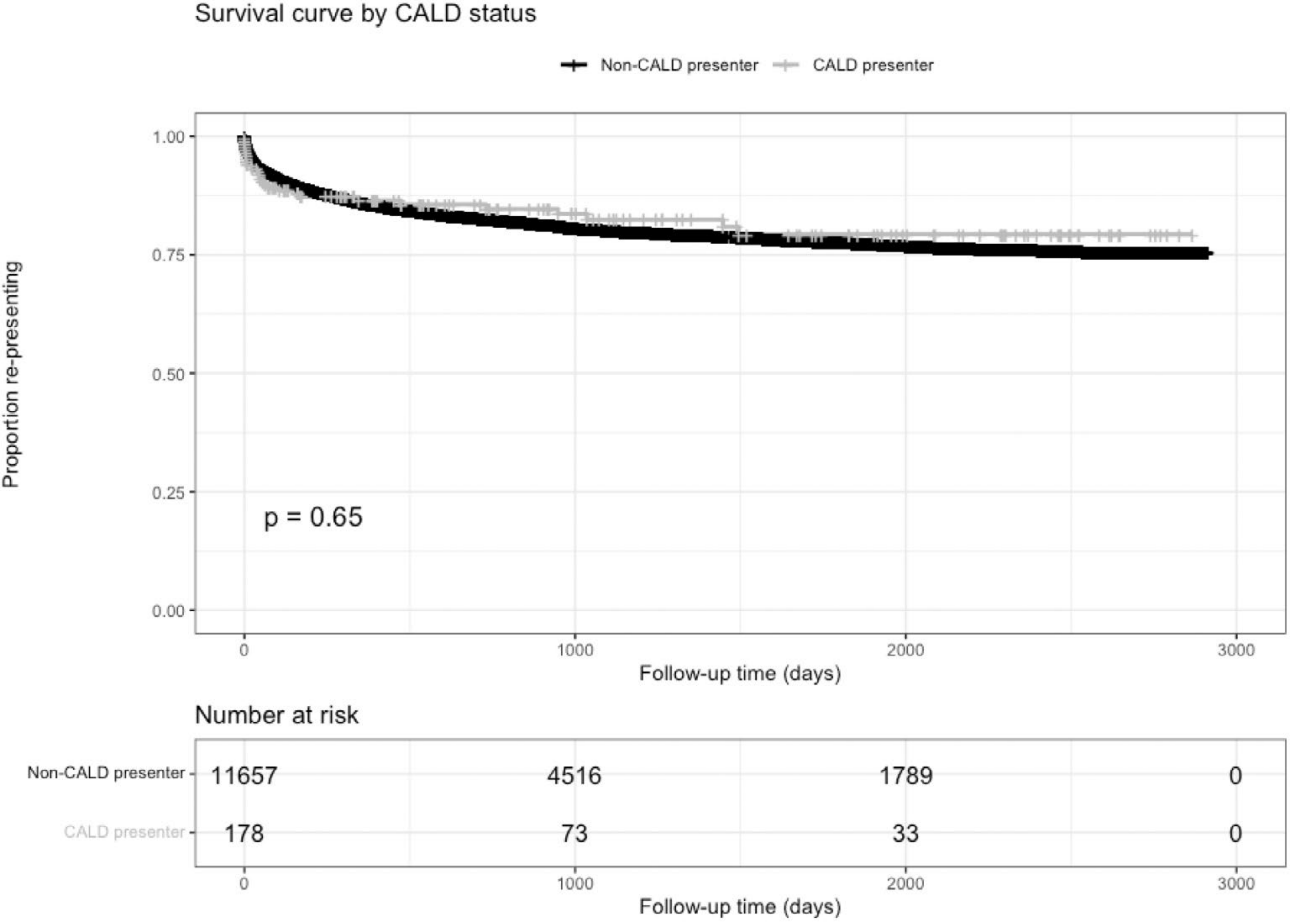
Kaplan–Meier curves by CALD status.

To examine the likelihood of first repeat presentation, log‐rank test and Cox's proportional hazards model were used. The log‐rank test found no difference in likelihood of representation between the CALD and non‐CALD group. In the first 3 months, 8.3% of the non‐CALD group had represented compared to 10.7% of the CALD group. In the first year, 14.2% of the non‐CALD group had represented, while 13.5% of the CALD group had represented (Figure 1).

The Cox proportional hazards model indicated that only gender and socioeconomic status were associated with the likelihood of representation (Table S1).

### 3.7 Extended Cox Regression Models for Multiple Presentations

CALD people had a 24% lower likelihood of representing to the ED at any time point within the study period, adjusting for previous presentations, based on the A–G model (Table 3).

**TABLE 3:** Extended multivariate cox regression model in examining factors associated with repeat self‐harm or suicide ideation presentations.

Effects	A–G model	*p*
	HR (95% CI)	
Age	1.00 (1.00–1.00)	<0.001
Gender (reference: females)		
Males	0.89 (0.85–0.93)	<0.001
Other	4.34 (3.24–5.82)	<0.001
CALD status	0.76 (0.61–0.96)	0.021
IRSAD decile	1.05 (1.04–1.06)	<0.001

## 4 Discussion

This study examined the patterns of ED presentations for self‐harm and suicide ideation over an 8‐year period between 12 January 2012 and 31 December 2019. It is the first in Australia to specifically examine differences in identified psychosocial and economic factors recorded in triage notes by CALD status. This study also compared service use between CALD and non‐CALD people and assessed differences in the likelihood of repeat presentations for self‐harm or suicide ideation.

Of the 15 606 presentations for self‐harm and suicide ideation, 202 (1.3%) were by CALD people, which is considerably lower than a similar study by Pham et al. (2023b) in which CALD presentations constituted 13% of total self‐harm hospital admissions, and may be explained by inconsistent recording of CALD status in triage notes. Many data sources used to report on the health of CALD populations do not collect any information on CALD status or may only collect information on one aspect (Australian Institute of Health and Welfare 2022). For example, language was the most common indicator recorded in the triage notes, yet this is only one aspect of CALD status. This focus on language may stem from its practical implications for service provision. Effective communication is an important part of therapeutic care, and there is evidence that people from CALD backgrounds are less likely to understand their condition and feel in control of their health compared to their non‐CALD counterparts following a trauma admission (Le et al. 2024). ED staff may benefit from having access to professionally trained medical interpreters for such presentations, which can lead to significant improvements in patient understanding and satisfaction, as well as nurse satisfaction (Bagchi et al. 2011). However, while acknowledging the importance of language for immediate communication needs, it may not capture the broader dimensions of CALD identity. Such an approach may risk oversimplifying the complexity of CALD experiences and needs, and other aspects of cultural, social and migration‐related factors that significantly influence health outcomes may be overlooked. These findings indicate the need for improved data collection methods at triage, such as the implementation of standardised recording processes and routinely asking individuals how they self‐identify.

Almost half of all CALD presentations occurred during business hours compared to one‐third of non‐CALD presentations. This may be explained by a lack of access to primary care among the CALD population (Rosano et al. 2013). CALD status may influence GP referral practices, directing asylum seekers to social services rather than mental health specialists (Ceuterick et al. 2020). Migrants tend to seek clinical care at crisis stage rather than an early intervention stage (Kirmayer et al. 2015). In this study, while CALD presentations may have initially been triaged as non‐urgent, further assessment may have revealed more acute needs, resulting in the greater frequency of ward admission that we observed. ED triage nurses often have a limited amount of time to determine an appropriate triage category (Broadbent, Moxham, and Dwyer 2014). Given many stigma‐related barriers to suicide disclosure among CALD populations (Flink et al. 2014), and the triage setting, which does not afford privacy (Richards et al. 2024), inadequate data may be collected at triage. It is necessary to evaluate whether these admissions reflect a genuinely higher need for care or if there are other contributing factors, such as potential disparities at triage or in outpatient support availability. A need for such specialised services may therefore drive CALD presentations to the ED rather than seeking other clinical services, such as a GP, that also operate during business hours. Future research should investigate how CALD people decide which clinical service to present to, and how clinical decisions are made once the individual attends a service. Additionally, Sterling et al. (2019) have previously suggested that nursing triage notes are an important communication tool for healthcare providers which can be used to provide informed and cohesive care by various staff, as well as inform hospital resource allocation which may reduce wait times and ED overcrowding. Both nurses and people who present to triage may benefit from a more private and secure triage environment (Veresova et al. 2024). This would allow people to be more forthcoming with information relating to their presentation that would enable more accurate triaging.

This study also found that CALD people may be 24% less likely to represent. This has also been observed in another analysis of Victorian hospital data (Stapelberg et al. 2020) and overseas (Farooq et al. 2021). Presentations by CALD people may be effectively addressed during their initial presentation, or there may be barriers deterring further help‐seeking. For example, a similar Australian study by Pham et al. (2023a) found that people from CALD backgrounds were less likely to engage mental health services compared to non‐CALD individuals (54% compared to 65%). To understand what influences representation in CALD people, future research should examine the reasons behind the decision‐making processes of CALD people post‐discharge and the continuity of care they receive or require.

The prevalence of occupational stressors, while low, was a point of difference between CALD and non‐CALD presentations, with such stressors mentioned three times more frequently in CALD presentations. Clements et al.'s (2019) analysis of hospital‐presenting self‐harm by middle‐aged people (aged 40–59) in the United Kingdom found that 17% of presentations had employment or study problems as precipitating factors, and 20% of presentations had financial problems. Another UK‐based analysis of hospital presentations for self‐harm by young people found a similarly high prevalence of employment or study problems, particularly among Black (25%), South Asian (25%) and other non‐White young (28%). Furthermore, an analysis of suicide deaths among psychiatric patients indicates that adverse life events such as serious financial difficulties and job loss may be more prevalent in the CALD population compared to the non‐CALD population (Tham et al. 2023). Given the variability in triage recording, it is likely that our analysis has underestimated the prevalence of such stressors, particularly in the CALD population who may be more likely to be impacted (Farooq et al. 2021). A holistic approach to assessment and treatment that includes consideration of occupational stressors is recommended.

These discrepancies between CALD and non‐CALD clinical management indicates a need to consider the mental health system more broadly, as highlighted by the Royal Commission into Victoria's Mental Health System (State of Victoria 2021), and how EDs should operate within this context. Victoria's mental health infrastructure has been critiqued for its inability to adequately serve those in need, marked by stigma, discrimination, power imbalances and a lack of community‐based services, leading to fragmented care and a neglect of mental health promotion and preventive strategies. Our findings contribute to this conversation by indicating possible areas for further research in treatment approaches, particularly given the distinct patterns of admission and representation rates among CALD people.

Our finding that CALD individuals are more frequently admitted to wards or mental health units suggests that there is an opportunity for aftercare services to bridge the gap in care continuity, ensuring these individuals receive support that could mitigate the severity of crises leading to hospital admissions. This is especially necessary given evidence that people from CALD backgrounds are less likely than non‐CALD people to be recommended further care after initial treatment (Bursztein Lipsicas et al. 2014; Farooq et al. 2021). Aftercare services have become an important part of the suicide prevention landscape, shown to lower rates of repeat self‐harm (Malakouti et al. 2021; Krysinska et al. 2016). However, evaluations of these services have yet to report substantial evidence of efficacy for CALD populations specifically (Kehoe et al. 2023; Mcgill et al. 2022). The commission has recommended expanding the Hospital Outreach Post‐suicidal Engagement (HOPE) programme, an assertive aftercare service designed to reduce repeat self‐harm (State of Victoria 2021). The differences observed in this study in terms of admission and representation indicate a need to examine the extent to which CALD people are provided referrals to aftercare services like HOPE, their experiences with such services and how this may influence future help‐seeking.

The development and implementation of culturally sensitive follow‐up procedures may support recovery post‐discharge (Hill, Halliday, and Reavley 2017). Given the gaps in understanding why CALD people may not seek recurring care from the ED, initiatives like New South Wales' feedback kiosks (NSW Health n.d.) which collect patient experiences in multiple languages and support process improvements may serve as a valuable model for enhancing cultural sensitivity in this context. Additionally, strengthening community outreach and mental health services (Salami, Salma, and Hegadoren 2019) and employment‐related services with a focus on employment conditions and prospects (Abdelkerim and Grace 2012) may improve CALD people's continuity of care.

### 4.1 Limitations

This study aggregated the study population into broad ‘CALD’ and ‘non‐CALD’ groups given the small proportion of presentations identified as CALD, as subgroup analyses would have been infeasible. A significant proportion of migrants who arrived between 2000–2001 and 2019–2020 were part of the skilled migration (60%) and family (39%) streams, subject to stringent eligibility criteria (Australian Institute of Health and Welfare 2022). This selection process may have led to the ‘healthy migrant effect’ being observed in this study, wherein the CALD group seems to have better health outcomes when data are presented in aggregate, and second‐generation migrants are not adequately accounted for. Research on self‐harm presentations in Victoria has previously found variations in self‐harm prevalence when disaggregated by country of birth (Pham et al. 2023b), and research from overseas has found differing prevalence of suicide ideation in second‐generation migrants compared to first‐generation migrants (Beutel et al. 2016). The aggregation in this study may have masked these intra‐group differences. Understanding how different CALD subgroups interact with healthcare services may lead to more tailored and individualised clinical approaches to such presentations. Future research may benefit from using linked data sets with a greater number of CALD variables, enabling more nuanced subgroups.

There is currently a lack of in‐depth research on suicide prevention in CALD populations, and a lack of comprehensive national statistics which disaggregate by CALD status. This study, due to variable triage recording, also likely undercounted the true proportion of CALD people in the study population. Both the CALD population and service providers may benefit from additional tools that allow for the collection of nuanced CALD data, including language preferences, country of origin and cultural background to also capture presentations from second‐generation migrants. This approach will facilitate improved public health surveillance on health status, risk factors and healthcare access to substantiate evidence‐informed policies and service delivery within clinical settings for the CALD population (Bozorgmehr et al. 2019). Country of birth information is part of the Victorian Emergency Minimum data set (State of Victoria 2023) However, it was not consistently recorded during triage in the database used in this study. While it is recognised that ED staff often face high workloads and time pressures (Richards et al. 2024), the importance of country of birth information for effective suicide prevention necessitates that staff are provided with the necessary support and resources to consistently record these data during the triage process.

There was also considerable variability in how gender/sex was recorded in the source data. To maintain consistency with the source data and acknowledge the potential variation between gender identity and biological sex, we used the combined term ‘gender/sex’. However, as a result, this may have unintentionally conflated biological sex and gender identity, which may not accurately represent the diverse spectrum of people who presented to the ED during the study period. Accurate and consistent recording of gender is as important as CALD documentation. This level of detail can aid service providers in providing personalised care that considers the intersectionality of age, gender and CALD status which has been explored previously (Bersani and Morabito 2020).

The analysis was restricted to presentations prior to the COVID‐19 pandemic, primarily because it is likely that the COVID‐19 pandemic would have distorted the findings, for example, by influencing both the accessibility of the ED for self‐harm and suicide ideation, but also the clinical management of those who presented. Deaths during this period were not captured, although we expect that about 1.2% of people with an index presentation for self‐harm or suicide ideation will have died by suicide during the follow‐up period (Murphy et al. 2023). We do not anticipate this will have significant impact on hazard ratio estimates. This analysis was also restricted to a single hospital, potentially missing repeat presentations to other hospitals or clinical services.

## 5 Conclusion

This study found that there is a need to improve the quality of data collection in EDs relating to CALD status, particularly during triage. Despite similar triage categorisations to non‐CALD presentations, CALD presentations were more likely to be admitted to a ward or mental health units. There is merit to understanding what drives these clinical decisions, the extent to which CALD people are connected to external supports post‐discharge, and how this relates to the lower likelihood of repeat presentations observed in the CALD group.

## 6 Relevance for Clinical Practice

This study emphasises the importance of routine recording of CALD status at the point of presentation to the ED. ED staff should be aware that people from CALD backgrounds may benefit from medically trained interpreters and may be experiencing occupational stressor. Additionally, CALD patients may benefit from ED‐facilitated connections to external supports. With findings indicating that CALD people are less likely to return to ED, ensuring appropriate referrals at discharge is important in maintaining continuity of care.

## Author Contributions

G.R. conceived the study idea, analysed data and drafted manuscript. K.W. provided guidance and support with data analysis. L.Z. provided support in developing the coding framework. J.R. and K.W. contributed to the interpretation of the results, and all authors contributed to the edit of the manuscript. All authors listed meet the authorship criteria according to the latest guidelines of the International Committee of Medical Journal Editors and are in agreement with the manuscript.

## Conflicts of Interest

The authors declare no conflicts of interest.

## Supporting information


Data S1.

## Data Availability

Research data are not shared.
