# Supplementary material for: Emergency Department Use Following Self‐Harm and Suicide Ideation: An Analysis of the Influence of Cultural and/or Linguistic Diversity Using Data From the Self‐Harm Monitoring System for Victoria (2012–2019)
Source: Int J Ment Health Nurs. 2024 Sep 9;34(1):e13411. doi: 10.1111/inm.13411 (PMC11751760; doi:10.1111/inm.13411)
Supplement: Supplementary file 1 — Data S1. [file INM-34-0-s001.zip › Supplementary Study 1.docx]

Supplementary File

Table S1. Variables used in analysis

|  | Type | Notes |
| --- | --- | --- |
| Demographic variables |  |  |
| Medical record number (MRN) | Categorical |  |
| Gender | Categorical | Coded as male, female, or intersex. Intersex presenters were excluded from tables to preserve privacy due to low cell counts. |
| Age | Continuous | Measured in years |
| Postcode | Categorical |  |
| Relative socio-economic advantage and disadvantage | Continuous | Deciles ranging from 1 (most disadvantaged) to 10 (most advantaged), derived from postcode |
| CALD status | Categorical | Post-processed binary variable ascertained through framework analysis |
| Presentation characteristics |  |  |
| Reason for presentation | Binary | Self-harm or suicide ideation |
| Time of arrival | Categorical | Early morning = 01:00-08:59  Business hours = 09:00-16:59  Night = 17:00-00:59 |
| Mode of arrival | Categorical |  |
| Emergency department length of stay | Continuous | Measured in minutes |
| Triage comment | Free-text |  |
| Triage category | Categorical | As per the Australasian Triage Scale, 1-5 |
| Receipt of doctor assessment | Binary |  |
| Receipt of mental health assessment | Binary |  |
| Time to mental health assessment | Continuous | Measured in minutes |
| Disposition | Categorical | Home, Ward, Mental Health Unit, Observation Unit, Left without being seen, Left before treatment completion, Other |

Inductive content analysis process:

**Inductive content analysis**

Each emergency department presentation’s corresponding triage comment was determined to be a “unit” of analysis. A level of familiarisation with the total dataset (N=15,606) was attained through the manual coding of CALD status described in section 3.4. Following this, a random sample of 10% of units was extracted from the broader dataset using Stata (StataCorp, 2017), resulting in a sample of 1,561 presentations, which was exported to NVivo (*NVivo 12*, 2017). The aim of this phase was to code themes or phenomenon identified within each triage comment to specific codes. This open coding process involved reading through each triage comment and annotating specific sections that were relevant to RQ2. For example, information about the presenter’s medical history, use of medication, and symptoms of mood disorders were coded. Notes within triage comments relating to vital signs or non-mental health related medical conditions were excluded from coding and subsequent analysis. Upon completion of this open coding process, the codes generated were grouped under higher-order headings to produce a coding framework.

**Pilot testing of coding framework**

To ensure inter-rater reliability and reproducibility of results, this coding framework was then pilot tested by two researchers who independently coded a randomly-selected subsample of 25% (n=3,900) of presentations, with frequent reviews to amend the framework. Cohen’s Kappa was then calculated for each individual code to measure the inter-rater reliability for that code. Codes with a Cohen’s Kappa of greater than 0.61 (substantial agreement (Landis & Koch, 1977)) were retained in the coding framework for subsequent coding of the entire sample (N=15,606).

Certain factors (i.e. borderline personality disorder, neurodevelopmental disorders, eating disorder, chronic pain, victimisation, grief, and use of helpline services) were removed from Table 2 due to low cell counts. Other factors (i.e. history and/or current alcohol or drug use, supported accommodation and housing circumstances, all personal relationships, and occupational and financial stressors) were collapsed due to low cell counts. The coding framework is presented in Figure S1.

**Figure S1.** Coding framework for the identification and coding of psychosocial and economic risk factors, organised into the broad categories of clinical/mental health, social, and service use.

**Recurrent event analysis**

To analyse all presentations by every individual, recurrent event analysis was also conducted. Recurrent event analysis takes a different approach to the original Cox proportional hazards model by making use of all events by each individual in the study population. Recurrent event analysis is also indicated for studies utilising multiple events by individuals over Poisson and negative binomial models as these approaches do not take into consideration the timing of events The Andersen-Gill (AG) model, also known as the counting-process model, assumes a common baseline hazard function for all events, and that the entire risk set is at risk for each repeat emergency department presentation(Amorim & Cai, 2015). The Breslow method was specified for handling ties, which is more efficient than the Efron and Exact methods when the number of tied events is small (Prasad & Rao, 2002). To apply each model, the coxph function in R was used (Therneau, 2023) . Proportional hazards assumption was tested for each predictor variable using the cox.zph function in R, and variables suspected of not meeting the proportional hazards assumption were checked graphically by plotting the scaled Schoenfeld residuals against time for the covariate. Covariates found to be in violation of the proportional hazards assumption were either accounted for by an interaction term or stratification in the final model.

**Survival analysis and first repeat presentation**

Table S2. Results of the Cox proportional hazards regression analysis of first repeat presentation

| Covariate | Univariate |  | Multivariate |  |
| --- | --- | --- | --- | --- |
|  | Hazard ratio (95% CI) | P-value | Hazard ratio (95% CI) | P-value |
| Age | 1.00 (1.00-1.00) | 0.240 | 1.00 (1.00-1.00) | 0.454 |
| Gender (reference: females) |  |  |  |  |
| Males | 0.99 (0.91-1.08) | 0.871 | 0.98 (0.89-1.07) | 0.656 |
| Other | **3.76 (1.69-8.41)** | **0.001** | **3.65 (1.64-8.16)** | **0.002** |
| CALD status | 0.92 (0.63-1.33) | 0.645 | 0.94 (0.64-1.39) | 0.768 |
| IRSAD decile | **1.06 (1.04-1.08)** | **<0.001** | **1.06 (1.04-1.08)** | **<0.001** |
