# Supplementary figures and images for: Emergency Department Use Following Self‐Harm and Suicide Ideation: An Analysis of the Influence of Cultural and/or Linguistic Diversity Using Data From the Self‐Harm Monitoring System for Victoria (2012–2019)
Source: Int J Ment Health Nurs. 2024 Sep 9;34(1):e13411. doi: 10.1111/inm.13411 (PMC11751760; doi:10.1111/inm.13411)

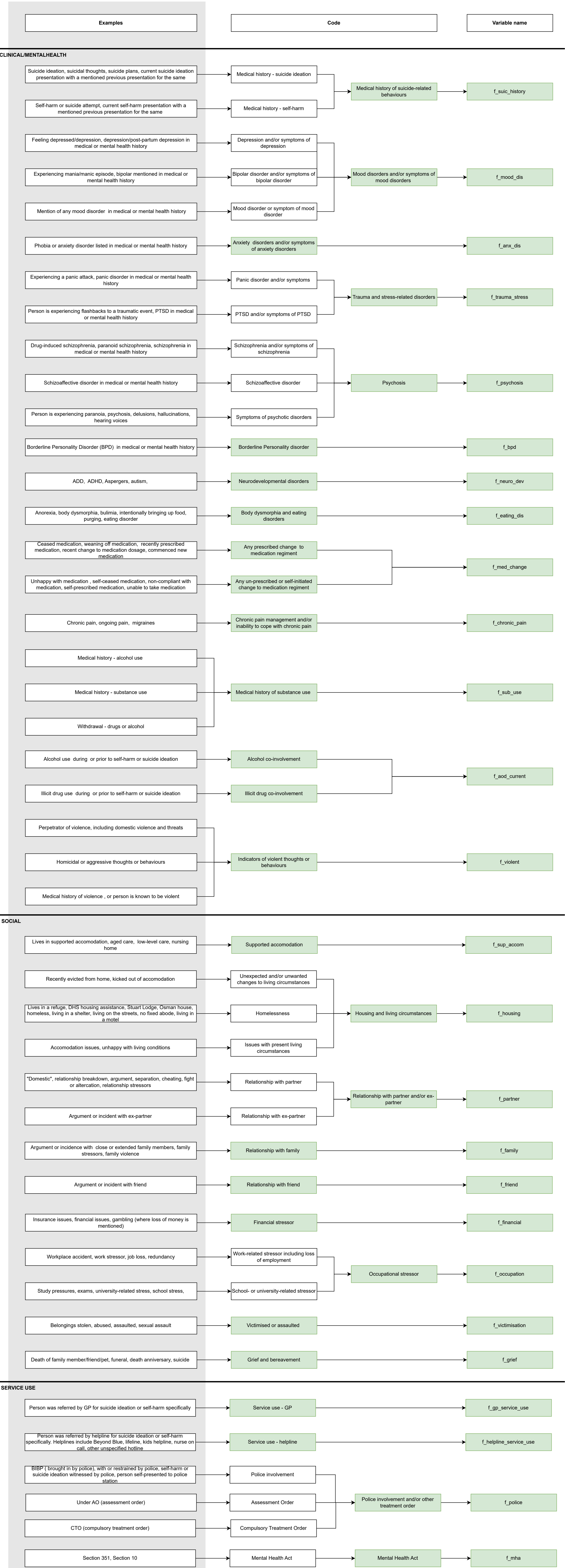

Supplement: Supplementary file 1 — Data S1. [file INM-34-0-s001.zip › Figure S1.pdf]
